# A Regularized Multi-Task Learning Approach for Cell Type Detection in Single-Cell RNA Sequencing Data

**DOI:** 10.3389/fgene.2022.788832

**Published:** 2022-04-13

**Authors:** Piu Upadhyay, Sumanta Ray

**Affiliations:** ^1^ B.P. Poddar Institute of Management and Technology, Kolkata, India; ^2^ Department of Computer Science and Engineering, Aliah University, Kolkata, India; ^3^ Health Analytics Network, Pittsburgh, PA, United States

**Keywords:** regularized multi-task learning(RMTL), cell type detection, scRNA-seq data, supervised learning, marker genes, manual annotation

## Abstract

Cell type prediction is one of the most challenging goals in single-cell RNA sequencing (scRNA-seq) data. Existing methods use unsupervised learning to identify signature genes in each cluster, followed by a literature survey to look up those genes for assigning cell types. However, finding potential marker genes in each cluster is cumbersome, which impedes the systematic analysis of single-cell RNA sequencing data. To address this challenge, we proposed a framework based on regularized multi-task learning (RMTL) that enables us to simultaneously learn the subpopulation associated with a particular cell type. Learning the structure of subpopulations is treated as a separate task in the multi-task learner. Regularization is used to modulate the multi-task model (e.g., *W*_1_, *W*_2_, … *W*_*t*_) jointly, according to the specific prior. For validating our model, we trained it with reference data constructed from a single-cell RNA sequencing experiment and applied it to a query dataset. We also predicted completely independent data (the query dataset) from the reference data which are used for training. We have checked the efficacy of the proposed method by comparing it with other state-of-the-art techniques well known for cell type detection. Results revealed that the proposed method performed accurately in detecting the cell type in scRNA-seq data and thus can be utilized as a useful tool in the scRNA-seq pipeline.

## 1 Introduction

There has been great interest recently in single-cell molecular profiling technologies, particularly when dealing with rare or highly specific cell types and states. Recent technological advances enabled us to process tens of thousands of cells per scRNA-seq experiment (Svensson et al., 2018). A fundamental step in the downstream analysis of single-cell data is to type the individual cells. The most popular and immediate approach is to identify the cell categories using unsupervised learning (Gribov et al., 2010; Kiselev et al., 2017), which is further analyzed to determine the cell categories. This way of analysis for annotating cells has been prevalent for identifying biologically coherent cell populations in scRNA-seq data so far (Cao et al., 2017; Fincher et al., 2018; Han et al., 2018; Plass et al., 2018).

Unsupervised (clustering) methods require manual annotation, which imply problems concerning the resolution of (sub-) types, manpower resources, and bias toward existing human knowledge. This step escapes the characteristic advantage of scRNA-seq data analysis because of the intervention of human manpower. Manual annotation depends on the prior knowledge of marker genes, which may be obtained from earlier bulk studies. For this, assigning the biological meaning of the cell clusters (groups) is not only a complicated task but also demands a huge amount of time. This problem becomes even worse when the number of cells and samples increases which surely prevents fast and reproducible annotation.

To overcome this problem, we need methods that automatically determine cell labels. With labeled data input, the *supervised learning* method (Alquicira-Hernández et al., 2018; Wagner and Yanai, 2018; Ma and Pellegrini, 2019; Pliner et al., 2019) can handle automatic and hassle-free cell type detection. Recently supervised approaches have gained popularity as they can determine cell types from the data, but the underlying molecular mechanisms are still not explored fully (Abdelaal et al., 2019).

There exist several methods which address the problem of cell type detection in scRNA-seq data using supervised (or unsupervised) approaches. Abdelaal et al. (2019) present an excellent review of the different supervised techniques for cell type detection. The task is to merely learn the cellular identities from annotated training data to predict the cell type. These approaches are relatively new compared with the large extent of methods available for addressing the computational challenges of a single-cell analysis. Pliner et al. (2019) have devised a method called Garnett for rapidly annotating cell types in single-cell RNA-seq data. Garnett operates on four steps: first, it defines a markup language to determine the cell types, and then, this language is processed by a parser that identifies representative cells bearing marker genes. In the third and fourth steps, it recognizes additional cells to each cell type based on their similarity to the representative and finally applies a classifier trained on one dataset. Wagner et al. (Wagner and Yanai, 2018) have introduced a new method based on a hierarchical machine learning framework that can construct robust cell type classifiers from heterogeneous scRNA-seq datasets. Also, Ma and Pellegrini (2019) have proposed a method ACTINN (Automated Cell Type Identification using Neural Networks), which utilized a neural network model with three hidden layers for training the dataset with predefined cell types. Here, predictions made for other datasets were based on trained parameters. The unbiased feature selection method is combined with machine learning classification to build a powerful method scPred (Alquicira-Hernández et al., 2018). It brings the advantage of dimensionality reduction and orthogonalization of gene expression values for accurately predicting the cell types. Most of the methods have proposed a model which trains the scRNA data for prediction or uses some feature selection techniques to reduce the dimension of the input data before clustering. So the training process solely depends on the whole data and ignores the crosstalk between multiple cell populations.

Here, we presented a framework based on the regularized multi-task learning (RMTL) approach for automatic cell type detection in the scRNA-seq datasets. The advantage of our model is that it can take multiple cell populations as the input, leveraging simultaneous learning of features. RMTL is already a well-explored field and is recently gaining popularity for solving numerous problems in the bioinformatics domain (Zhang et al., 2018; Dizaji et al., 2020; Wang et al., 2020). The performance of learning will increase if we learn from multiple interrelated tasks (Baxter, 1997). The multi-task learning approach can also tackle the overfitting issue. The crucial task here was to find out the shared parameters for identifying the relationship with common features among the tasks (Singh et al., 2019). Our model took samples of different cellular identities as reference input data and predicted cell types from query datasets. Here, we hypothesized that the biological information of cell samples coming from several cell types was related in some way, and for this reason, we have to learn all cell samples simultaneously using multi-task learning. Here, *L*_2,1_ regularization is used to smooth the loss function, thereby minimizing the complexity of the model. We have compared the proposed method with four state-of-the-art, widely used cell type detection tasks for scRNA-seq data. The results showed the proposed method outperformed the other in automatically detecting the cell types.

## 2 Materials and Methods

### 2.1 Dataset Description and Preprocessing

The following datasets are used for the experiments:

1. CBMC (Stoeckius et al., 2017): The datasets of cord blood mononuclear cells produced by CITE-seq contain 8,617 cells in the RNA UMI matrix, downloaded from https://www.ncbi.nlm.nih.gov/geo/under the accession no. GSE100866. This transcriptome comprises 13 cell types over 7,985 transcriptomes.
2. Goolam (Goolam et al., 2016): It has been constructed from mouse embryos in five stages/levels (2-cell, 4-cell, 8-cell, 16-cell, and blast). In total, 124 cells with 41,428 genes were present, downloaded from https://www.ebi.ac.uk/arrayexpress/files/E-MTAB-3321/E-MTAB-3321.processed.1.zip
3. Melanoma (Tirosh et al., 2016): The dataset has been downloaded from https://www.ncbi.nlm.nih.gov/geo/under the accession no. GSE72056 and contains 23,686 genes with 4,645 cells. Details of datasets, after preprocessing, are given in Table 1. We adopted the standard pipeline of Seurat v3. (Stuart et al., 2019) for the preliminary analysis and preprocessing, particularly quality control, cell and gene filtering, and normalization.
4. Yan (Yan et al., 2013): This is a human preimplantation embryo and embryonic stem cell dataset. The average total read count in the expression matrix is 25,228,939 reads. There are seven cell types, including the labeled 4-cell, 8-cell, zygote, late blastocyst, and 16-cell type [GEO under the accession no. GSE36552]. Experiments are performed by high-throughput sequencing. The authors used the *de novo* transcriptome reconstruction software Trinity (Grabherr et al., 2011) and the eukaryotic genome annotation tool PASA to perform the *de novo* assembly of reads.
5. Klein (Klein et al., 2015): This dataset was generated by the droplet barcoding method with an average total read count of 20,033.40 reads in the expression matrix. A total of eight single-cell datasets are submitted: three for mouse embryonic stem (ES) cells (one biological replicate and two technical replicates); three samples following LIF withdrawal (days 2, 4, and 7); one pure RNA dataset (from human lymphoblast K562 cells); and one sample of single K562 cells. The dataset was downloaded from GEO under the accession no. GSE65525. The dataset contains 24,175 number of genes and 2,717 number of cells with four cell types. Cells are captured and barcoded in nanoliter droplets with high capture efficiency.
6. PBMC68k (Zheng et al., 2017): The dataset is downloaded from the 10x Genomics website https://support.10xgenomics.com/single-cell-geneexpression/datasets. The data are sequenced on Illumina NextSeq 500 high output with 20,000 reads per cell.

**TABLE 1:** Details of the used dataset.

Dataset	No. of cells	No. of genes	No. of cell types
CBMC	7,895	2,000	13
Goolam	124	40,315	5
Melanoma	5,038	3,546	8
PBMC	32,738	68,793	11
Yan	20,514	90	7
Klein	24,175	2,717	4

#### 2.1.1 Preprocessing

In the initial step, we have collected a single-cell RNA count matrix from different sources. Columns of these matrices contain cell/sample information, and genes are represented row-wise. The RNA counts are organized as a matrix *M*_*cl*×*ge*_, where *cl* is the number of cells and *ge* is the number of genes. Each element [*M*]_*ij*_ represents the count of the *i*th cell and the *j*th gene. If more than a thousand genes are expressed (non-zero values) in one cell, then the cell is termed as good. We assumed one gene is expressed if the minimum read count of it exceeds 5 in at least 10*%* of the good cells. The data matrix *M* with expressed genes and good cells is normalized using a linear model and normality-based normalizing transformation method (Linnorm) (Yip et al., 2017). The resulting matrix (*M*_*cl*′×*ge*_’) is then log_2_ transformed by adding one as a pseudocount.

### 2.2 A Short Description on Multi-Task Learning

Baxter et al., 1997 first introduced the concept of multi-task learning through the theoretical learning of multiple task and describes the multi-task sampling usage for the Bayesian model. This model was utilized to know how much information is required by individual task to learn. Baxter et al., 2000 established the concept of inductive biases for searching optimal hypothesis in the environment of multiple related task. Ben and Schuller, 2003 have utilized generalized VC dimension to derive bounds for each task while assuming that the learning tasks are related.

The main assumption behind the usage of multi-task learning (MTL) is that the tasks that comes under different types of learning are related to each other. Example of learning tasks may be supervised learning (e.g., classification, regression), unsupervised learning (e.g., clustering), reinforcement study, semi-supervised learning, and many more. Among all the learning tasks, all tasks or a subset of tasks are related to each another. The motivation behind that simultaneous learning of related and multiple tasks leads improved performance rather than learning a single task. The primary intention of using MTL is to enhance the generalized performance between related tasks.

The main idea is that given Z learning task, assuming that the datasets for these tasks are coming from same space of X × Y, the conditional distribution of the response variable Y; Y_z_|X_z_ are related, where X is the explanatory variable of all the Z tasks. In particular, given z learning tasks : $\tau_{i=1}^{z}$ , each task having n data points: (x_1z_, y_1z_), (x_2z_, y_2z_), … (x_nz_, y_nz_), where each data point is coming from a distribution P_z_ on X × Y. Here P_z_s are different for each task, however MTL assumes that these are related. Now the aim is to learn z functions f_1_,f_2_ … f_z_ each of which corresponds to a learning task as: f_z_(x_iz_)=y_iz_. For z=1 the problem reduces to single-task learning. Several setups may also be possible, such as when the input data x_iz_ are same for all task, but output value y_iz_ differs from each other. The other scenario may be the case of having the same output y_it_ for different inputs x_it_, which corresponds to the problem of integrating information from heterogeneous databases Ben-David et al., 2002.

### 2.3 Description of the Proposed Methodology

**FIGURE 1:**
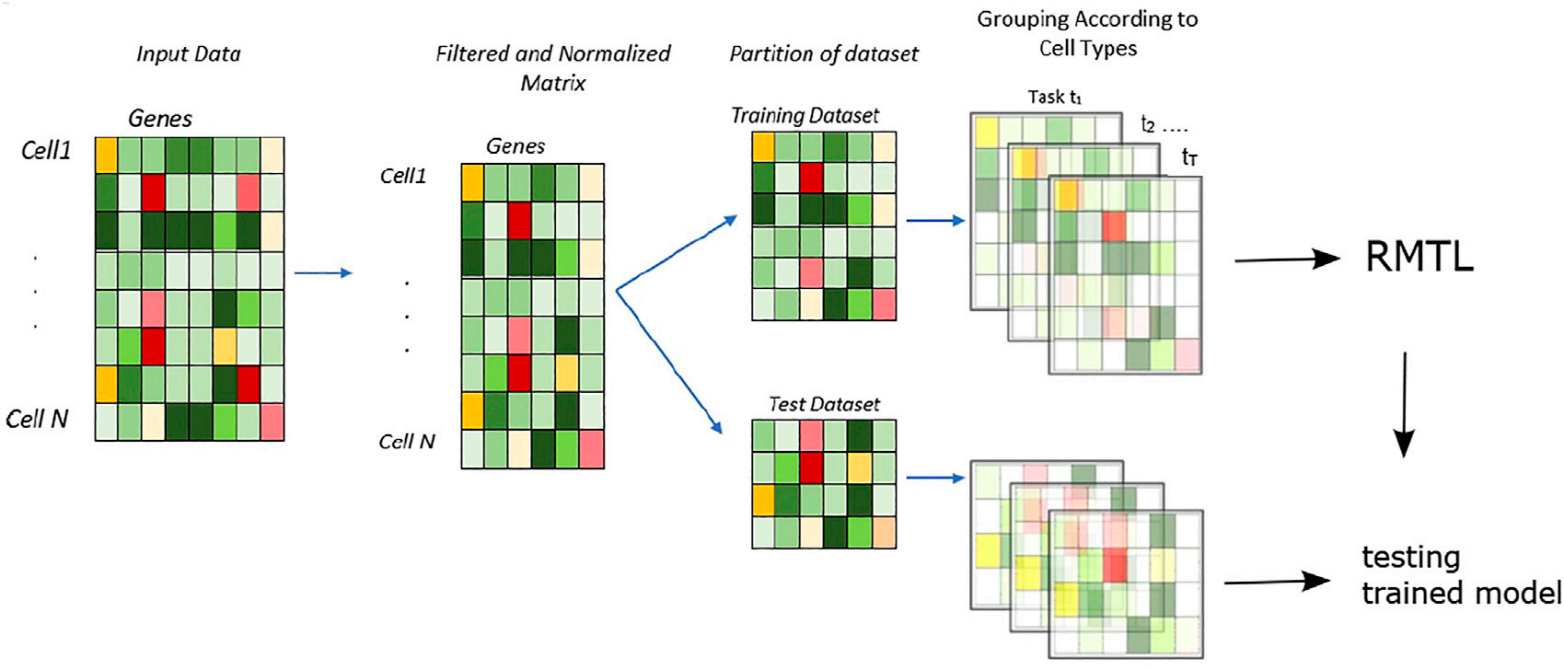
Workflow of the methodology: the proposed approach for cell type identification—the data are randomly divided into training and test sets. The cell types present in training sets are used to train the multi-task learning classifier with cross-validation. Then, the learnt model is tested with test datasets, and accuracy is measured with a confusion matrix.

We have proposed a supervised model which leverages the characteristics of the regularized multi-task learning algorithm for efficiently identifying cell types present in the scRNA-seq datasets. The overall analysis is shown in Figure 1.

#### 2.3.1 Multi-Task Learning for Cell Type Prediction

A regularization-based approach (Evgeniou and Pontil, 2004) is proposed to solve the MTL problem, where the regularization functions are minimized in an analogous way to SVM which is used in single-task learning. All the algorithms more or less try to minimize the following function:

$$\overset{t}{\underset{i=1}{\sum }}L\left(W_{i},C_{i}|X_{i},Y_{i}\right)+\lambda_{1}\omega \left(W\right)+\lambda_{2}\|W{\|}_{2}^{F}$$

where *L* (◦) represents the loss function, *ω* represents the cross-task regularization, and *λ*_1_ and *λ*_2_ are positive regularization parameters. *λ*_1_ signifies the strength of relatedness of all tasks and is estimated through a cross-validation procedure, whereas *λ*_2_ is to introduce the penalty of the quadratic form of *W*.

The tasks in our case are to learn different subpopulations (*S*_1_, *S*_2_, … *S*_*T*_) of *T* cell types. Each task can be represented as *S*_*i*_ = {(*X*_*i*_, *Y*_*i*_), *X*_*i*_ ∈ *R* ^*n*×*p*^, *Y*_*i*_ ∈ {1,−1}^*n*^}, where *n* represents the number of samples, and p represents the number of genes in scRNA-seq data. Assuming the generalized linear model *Y*_*it*_ = *f*_*t*_ (*W*_*t*_.*X*_*it*_) = *f*_*t*_ ((*W*_0_ + *V*_*t*_). *X*_*it*_), for each t ∈ 1,2, T,“.” represents the standard dot product in *R* ^*d*^, where *W*_*t*_ = *W*_0_ + *V*_*t*_. Here, the vector *W*_*t*_ corresponds to the linear model for each task. *V*_*t*_ controls the task relatedness. Here, we have utilized the RMTL framework, developed to estimate the parameters *W*_0_ and *V*_*t*_:

$$min_{W_{0},V_{t}}\overset{T}{\underset{t=1}{\sum }}\overset{n}{\underset{i=1}{\sum }}\left(Y_{it}-f_{t}\left(W_{t}.X_{it}\right)\right.+\lambda_{1}\|W_{0}{\|}^{2}+\lambda_{2}\overset{T}{\underset{t=1}{\sum }}\|V_{t}{\|}^{2}$$

where the first summation represents the loss over all tasks for each data point, and *λ*_1_ and *λ*_2_ positive regularization parameters trade-off between fitting the data and smoothness of the estimate.

Primarily, the input matrix is randomly partitioned into two parts: training and test sets (Figure 1). In total, 80% of the data are randomly selected for training purposes, and the rest 20% are used for testing. In this model, the number of tasks are presented as *t* ∈ 1, 2, … *T*; here, each task represents the learning of expression data from individual cells.

Here, the reference data are the scRNA-seq expression matrix over all the cellular identities. We applied the proposed model in the reference data for training. We tested the accuracy of our model in the query data. The query dataset is, of course, excluded from the reference for training. Therefore, we used completely independent data as references and tested the model with independent data. The accuracy of the model is calculated with the percentage of correctly identifying cell types in test data.

## 3 Results

### 3.1 Comparisons of the Proposed Method With State-Of-The-Art Methods

#### 3.1.1 Description of State-Of-The-Art Methods

We have compared the proposed method with the current state-of-the-art techniques in supervised learning–based single-cell typing. Supervised learning is advantageous over unsupervised learning (clustering) because it automatizes the cell typing procedure instead of manual annotation. Garnett (Pliner et al., 2019) annotates cell types in single-cell RNA-seq data by defining a markup language to specify cell types that are subsequently processed by a parser that identifies representative cells bearing marker genes. New cells are assigned to cell types based on their similarity to representative cells. An alternative work presents a hierarchical machine learning framework that yields robust cell type classifiers trained on heterogeneous scRNA-Seq datasets (Wagner and Yanai, 2018). ACTINN (Automated Cell Type Identification using Neural Networks), presents a neural network model with three hidden layers, which are trained and used for prediction in the usual way (Ma and Pellegrini, 2019). scPred combines an unbiased feature selection method with standard machine learning classification, where dimensionality reduction and orthogonalization of gene expression values prove advantageous to accurately predict cell types (Alquicira-Hernández et al., 2018). CHETAH builds a hierarchical classification tree from the reference (training) dataset and classifies unknown samples by computing the correlation between genes that discriminate the test cell from the reference dataset (de Kanter et al., 2019). In this work, we have considered 1) *scPred* (Alquicira-Hernández et al., 2018), 2) *ACTINN* (Ma and Pellegrini, 2019) (Automated Cell Type Identification using Neural Networks), 3) *CHETAH* (de Kanter et al., 2019), and 4) *Garnett* (Pliner et al., 2019) as the state-of-the-art methods for comparisons.

#### 3.1.2 Training and Test Data

Each scRNA-seq dataset is divided into training and test data at a ratio of 8:2. The performance of each competing method is evaluated by determining the average test accuracy and the corresponding standard errors over 100 runs. To know how the different methods react to reducing the training data, data were subsampled at rates ranging from 20 to 100% in steps of 20% prior to training. For each of the 100 runs, random subsamples were drawn independently.

#### 3.1.3 Evaluation

Table 2 displays the corresponding mean accuracy with the standard deviation for 100 independent runs. It is evident that the proposed method outperformed the state-of-the-art method with respect to the accuracy score. It can be noticed that although for a small number of training samples, the performance is not much impressive, and the proposed method outperformed others when sufficient samples are available for training.

**TABLE 2:** Prediction accuracy on test data for different datasets and different methods. Results refer to integrated data representations for all datasets. The test accuracy is displayed as mean ± standard deviation, referring to 100 randomly initialized training runs. Percentages refer to the relative amount of training data used during training. Maximum values for means and minimum values for standard deviation of the test accuracy are highlighted in bold.

Method	40%	60%	80%	100%	40%	60%	80%	100%
	*CBMC*				*Klein*			
scPred	71.87 ± 0.29	77.92 ± 0.20	84.81 ± 0.09	90.26 ± 0.01	61.25 ± 0.31	69.32 ± 0.20	75.46 ± 0.21	78.71 ± 0.10
ACTINN	70.78 ± 0.25	**81.80 ± 0.18**	**92.29 ± 0.15**	96.03 ± 0.10	62.14 ± 0.35	68.42 ± 0.31	73.81 ± 0.22	77.85 ± 0.10
CHETAH	66.97 ± 0.11	73.71 ± 0.15	87.91 ± 0.10	94.34 ± 0.01	68.91 ± 0.30	72.34 ± 0.15	77.63 ± 0.11	81.29 ± 0.10
Garnett	69.75 ± 0.28	79.68 ± 0.19	85.59 ± 0.19	96.01 ± 0.18	64.81 ± 0.30	**75.75 ± 0.31**	**77.84 ± 0.18**	81.61 ± 0.10
RMTL	**70.23** **±** **0.03**	80.53 ± 0.01	89.58 ± 0.06	**97.05 ± 0.01**	**69.31** **±** **0.03**	70.34 ± 0.03	76.85 ± 0.02	**81.72 ± 0.02**
	*Melanoma*				*PBMC68k*			
scPred	60.29 ± 0.31	68.91 ± 0.30	**79.10 ± 0.25**	**83.57 ± 0.19**	65.86 ± 0.05	69.87 ± 0.17	71.48 ± 0.13	78.25 ± 0.10
ACTINN	62.30 ± 0.43	67.51 ± 0.35	73.90 ± 0.19	78.31 ± 0.09	63.21 ± 0.31	73.91 ± 0.29	74.56 ± 0.19	81.29 ± 0.19
CHETAH	62.38 ± 0.20	65.19 ± 0.10	77.35 ± 0.11	81.82 ± 0.07	61.37 ± 0.0.28	63.45 ± 0.22	72.71 ± 0.19	81.19 ± 0.10
Garnett	66.27 ± 0.05	68.31 ± 0.05	71.81 ± 0.01	79.72 ± 0.01	**68.50** **±** **0.10**	72.53 ± 0.10	77.81 ± 0.05	82.10 ± 0.01
RMTL	**67.18** **±** **0.05**	**69.31 ± 0.05**	70.61 ± **0.01**	82.12 ± 0.01	64.50 ± 0.10	**74.53 ± 0.10**	**77.92 ± 0.05**	**82.73 ± 0.01**
	*Goolam*				*Yan*			
scPred	**68.29** **±** **0.31**	**72.11 ± 0.30**	77.10 ± 0.25	86.57 ± 0.19	77.16 ± 0.05	**85.57 ± 0.27**	**90.68 ± 0.23**	97.25 ± 0.10
ACTINN	62.10 ± 0.33	64.51 ± 0.35	68.90 ± 0.19	70.31 ± 0.09	76.11 ± 0.21	88.91 ± 0.29	90.16 ± 0.19	94.29 ± 0.01
CHETAH	60.38 ± 0.20	66.29 ± 0.10	72.15 ± 0.11	85.82 ± 0.07	71.37 ± 0.0.28	78.45 ± 0.22	83.71 ± 0.29	92.29 ± 0.10
Garnett	66.27 ± 0.05	72.21 ± 0.05	**78.61 ± 0.01**	83.72 ± 0.01	75.50 ± 0.10	82.53 ± 0.10	85.81 ± 0.05	92.10 ± 0.01
RMTL	63.27 ± 0.05	68.31 ± 0.05	72.41 ± 0.01	**86.72 ± 0.01**	**77.50** **±** **0.10**	80.33 ± 0.10	87.81 ± 0.05	**97.10 ± 0.01**

The amount of training data used during the training phase (in percentage).

The bold values represent the amount of training data used during the training phase (in percentage).

**FIGURE 2:**
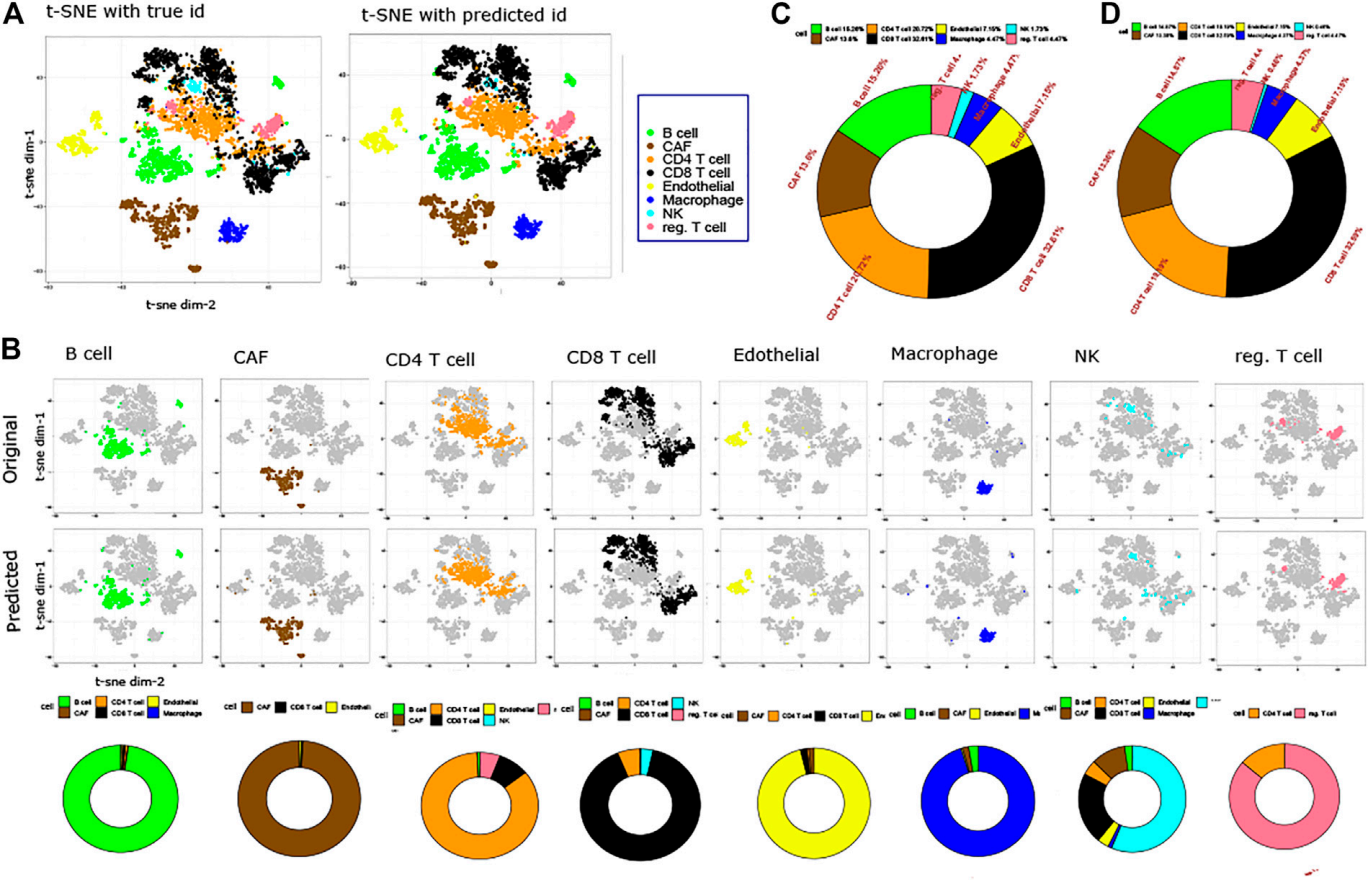
Prediction results on the Melanoma dataset. **(A)**. Two-dimensional t-SNE plot representing original and predicted labels of melanoma data. **(B)**. t-SNE visualization of original and predicted labels for individual cells. Each column shows three figures: the first and second one represent original and predicted labels in a two-dimensional t-SNE embedding, while the third one shows a donut plot proportion of true-positive and false-positive samples of the predicted labels. **(C)**. Proportion of original cell types within the data is shown in a donut plot. **(D)**. Proportion of predicted labels is shown in a donut plot.

To visualize the original and predicted labels, we performed t-SNE-based embedding of the datasets. Figure 2 shows two-dimensional t-SNE-based embedding of melanoma data with its original and predicted labels. The predicted labels are obtained from the trained model. Figure 2A represents the t-SNE embedding of melanoma data for original and predicted labels. Figure 2B demonstrates the comparison between the predicted and original labels for each individual cell. Of note, some minor cells such as macrophages (4.47% of the total cells), endothelial cells (7.15% of the total cells), and NK cells (1.73% of the total cells) which come with little samples are also correctly predicted by the proposed method. To show the false positive of the prediction, bottom figures of Figure 2B show a set of donut charts that represent the misclassification of cells. It is evident from the figure that in most of the cases (except Treg cells), the false-positive rate is extremely low. Figures 2C,D demonstrate the percentage of original and predicted cell types present in the melanoma data in two donut charts, respectively. It can be noticed that CAF (13.6% of the original cell samples and 13.6% of the predicted cell samples), CD8 T (32.61% of the original cell samples and 32.59% of the predicted cell samples), CD4 T (20.72% of the original cell samples and 19.19% of the predicted cell samples), and endothelial (7.15% of the original cell samples and 7.15% of the predicted cell samples) cells are predicted with utmost accuracy. A similar conclusion can be drawn from the CBMC data classification. Supplementary Figure S1 shows the classification results in detail.

### 3.2 Proposed Method Can Identify Poorly Covered Cell Accurately

In this study, our aim was to see how the poorly covered cell types, which generally come with little training data, can be detected by the competing methods. For this, we have applied the trained model on test data and computed the recall and precision score for the samples of a particular cell type. Considering the heterogeneous distribution of cells, we performed this experiment on CBMC and in Melanoma datasets. For other datasets, cells have a sufficient proportion of samples, such as for Klein, and the proportion of samples of four cell types are type-1 (11.15%), type-2 (25.13%), type-3 (29.37%), and type-4 (34.33%). Table 3 shows the results for these three datasets. It is evident from the table that the cell type with the small number of samples (such as pDCs and DC for CBMC, NK and Treg cells for Melanoma, and dendritic, CD34^+^, CD14^+^ cells, and monocytes for PBMC68k) proposed a method that outperformed the other models in terms of correct predictions.

**TABLE 3:** Table shows the percentage of correct prediction for all the competing models on CBMC and Melanoma datasets.

Cell type	#Samples present in the dataset	Methods									
		RMTL		Garnett		scPred		ACTINN		CHETAH	
		Recall	prec	recall	prec	recall	prec	recall	prec	recall	prec
CBMC											
Eryth	105	94	93.1	92.6	86.5	81.8	78.7	93.9	91.8	89.8	84.7
NK	1,089	87.77	94.8	88.7	89.7	84	80.8	89.2	88.1	86	80.3
CD14^+^ mono	2,293	97.7	99.1	98.8	98.1	93.8	92.7	99	97.5	97.8	96
MK	96	92.1	89.6	85.3	81.2	78.6	71.9	88.1	85.3	83.7	78.9
CD34^+^	119	89.04	88.8	87.8	82.9	82.3	81.8	96.4	91.5	84.3	80.2
DC	70	91.1	90.8	82.8	79.8	79	78.6	92.7	90.6	82	80
Memory CD4 T	1,781	97	95.1	97.6	91.6	90	91.7	97	96.4	93.9	90.8
CD8 T	273	90.2	89.7	86	81.8	83.8	79.0	90.4	82.8	89	81.2
CD16^+^ mono	230	87.7	88.5	90	86.8	80.7	78.4	85.8	80.9	81.8	80
B	350	93.3	91.07	92.7	90.5	88.6	88.1	96	94.6	92.7	89.2
T/mono doublets	182	92.7	91.5	88.7	81.3	85.1	81.7	97.2	90	91.8	88.3
PDcs	49	91.8	90	93.2	85.5	81.2	75.2	93	88.8	86.5	78.6
Naive CD4 T	1,248	98.2	96.6	97.8	89	88.3	81.8	98	86.7	88	79.1
Melanoma											
B cells	729	96.5	93.6	91.8	89	88.3	81.8	95	86.7	88	79.1
Macrophages	225	89.3	88.1	81.7	87.3	86.8	89.1	88.7	81.5	84.7	83.9
NK	87	83.9	85.6	80.9	84.3	80.1	81.9	82.7	82.3	83.6	85.1
CAF	685	95.8	97.7	91.7	95.8	93.2	94.4	96.8	96.9	95.7	93.4
Endothelial cells	360	90.3	92.8	88.8	90.6	90.1	90.5	91.5	91.7	89.4	90.2
CD4 T cells	1,044	98.1	97.3	96.1	97.9	95.8	98.4	98.2	95.1	92.9	95.8
CD8 T cells	1,643	97.1	97.7	95.6	96.3	96.8	92.9	96.1	96.0	93.9	97.1
Treg cells	225	90.6	89.1	88.2	88.1	89.0	86.8	89.2	86.9	87.1	88.9

### 3.3 Stability Performance

**FIGURE 3:**
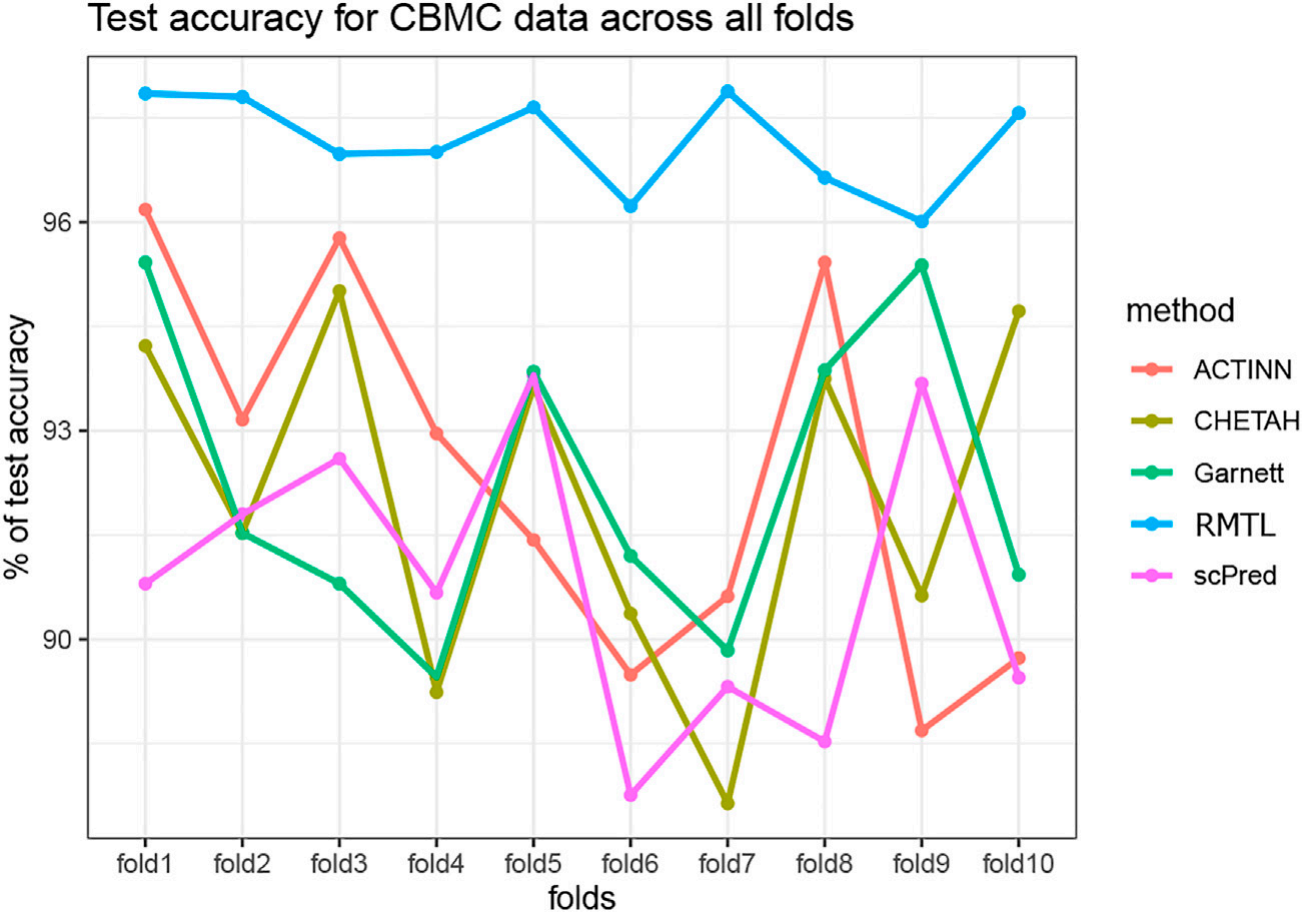
Test accuracy across all the folds for the five competing methods on CBMC data.

To compare the stability in the performance of the four competing methods, we have carried out a 10-fold cross-validation analysis for all the datasets. In each fold, we randomly divided the training data as training: validation in the ratio 9:1 and computed the validation accuracy. The process was repeated 100 times for each fold. Thus, in each fold we obtained 100 validation accuracy and one test accuracy for each of the competing method. The medians of the validation accuracy were compared with a Wilcoxon rank-sum test across the folds. Table 4 shows the *p*-value for all the competing methods across all the datasets. Although all methods produce stable results with low *p*-values, nevertheless, the proposed method showed a more stable performance among the other methods. Figure 3 also shows the test accuracy for all the methods across the folds for the CBMC dataset (see Supplementary Material for the results of other datasets). From Table 3 and Figure 3, it is evident that the proposed method outperformed the others for producing stable results.

**TABLE 4:** *p*-value obtained from the Wilcoxon rank-sum test for the five competing methods.

Method	CBMC	Klein	Melanoma	PBMC68k	Goolam	Yan
scPred	2.01E-02	1.09E-03	3.87E-02	4.6E-02	1.87E-03	1.98E-02
ACTINN	1.08E-02	2.8E-03	2.08E-02	2.96E-02	1.09E-03	1.87E-02
CHETAH	1.78E-03	1.6E-02	1.78E-02	2.98E-02	1.98E-03	2.89E-02
Garnett	2.86E-02	1.76E-02	2.10E-02	1.76E-02	1.65E-03	1.87E-02
RMTL	1.05E-03	2.56E-03	1.89E-03	1.87E-02	1.09E-03	1.07E-03

### 3.4 Execution Time

All experiments were carried out on a Linux server having 50 cores and a *X*86_64 platform. To compare the execution time of the competing methods, we performed an analysis. Four simulated data [using splatter (Zappia et al., 2017)] are generated by varying the number of cells and classes as follows: 500 cells with two classes, 1,000 cells with three classes, 1,500 cells with four classes, and 2,000 cells with five classes. All the simulated datasets are generated keeping equal group probabilities, 2,000 number of features with a fixed dropout rate as 0.2. In each case, the runtime is compared with different competing methods (see Table 5).

**TABLE 5:** Execution time (in minute) for the five competing methods.

Dataset	#Feature	# Cell	# Class	Execution time (in min)				
				scPred	ACTINN	Garnett	CHETAH	RMTL
Data 1	2,000	500	2	2	1	2	3	1
Data 2	2,000	1,000	3	4	1	4	7	1
Data 3	2,000	1,500	4	10	5	10	11	4
Data 4	2,000	2,000	5	15	10	16	16	9

## 4 Discussion

Our proposed methodology addresses the cell type prediction issue by having a vigorous multi-task learning model to predict the cell types efficiently. This cell type detection is very crucial in many applications of single-cell RNA sequencing data. The results demonstrated that the *L*_21_ regularization technique helps in jointly learning the features of cell types.

In experiments referring to six different data sets CBMC (Stoeckius et al., 2017), Goolam (Goolam et al., 2016), Melanoma (Tirosh et al., 2016), PBMC (Zheng et al., 2017), Yan (Yan et al., 2013), and Klein (Klein et al., 2015), we evaluated how the proposed method was performed in comparison with other methods (scPred (Alquicira-Hernández et al., 2018), ACTINN (Ma and Pellegrini, 2019), CHETAH (de Kanter et al., 2019), and Garnett (Pliner et al., 2019). The proposed method outperformed the other methods on all datasets utilized in this study. It also outperformed the others in terms of economic use of training samples. For example, for the datasets Klein, CBMC, Melanoma, and Yan, 40% of the training samples are enough to obtain more than 65% accuracy. Of note, exactly these advantages meant landmark arguments for regularized multi-task learning also in their original application.

In summary, we provided a new method that implemented the latest advances in machine learning for the purposes of typing single cells on basic heterogeneous single-cell RNA sequencing data. We have demonstrated that the theoretical promises can indeed be leveraged. In this study, we argued to have pushed the limits in single-cell typing by a non-negligible amount.

We concluded by acknowledging that also our method, of course, leaves room for improvement: various open problems are still awaiting their solutions. For example, one challenge is the fact that our method, by virtue of being a supervised approach, requires cell annotations prior to classification. Although an automated approach is much needed over approaches that require manual intervention at some point, however, actionable annotations need to be provided prior running the method.

## Data Availability

The original contributions presented in the study are included in the article/Supplementary Material, further inquiries can be directed to the corresponding author.
