# Supplementary material for: A Regularized Multi-Task Learning Approach for Cell Type Detection in Single-Cell RNA Sequencing Data
Source: Front Genet. 2022 Apr 13;13:788832. doi: 10.3389/fgene.2022.788832 (PMC9043858; doi:10.3389/fgene.2022.788832)

A. Donut plot for some cell types of CBMC Dataset

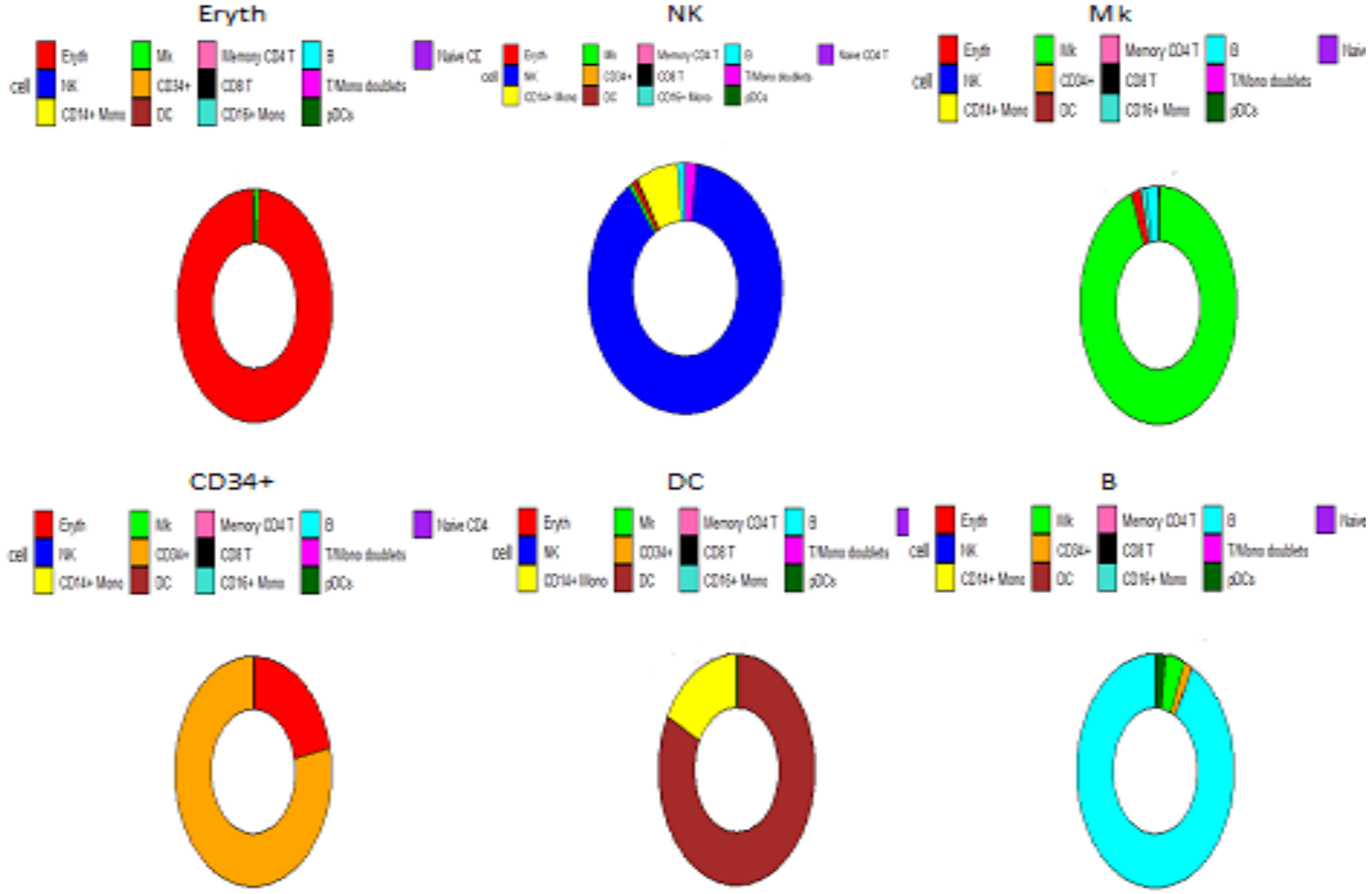

B. Donut plot for some cell types of Melanoma Dataset

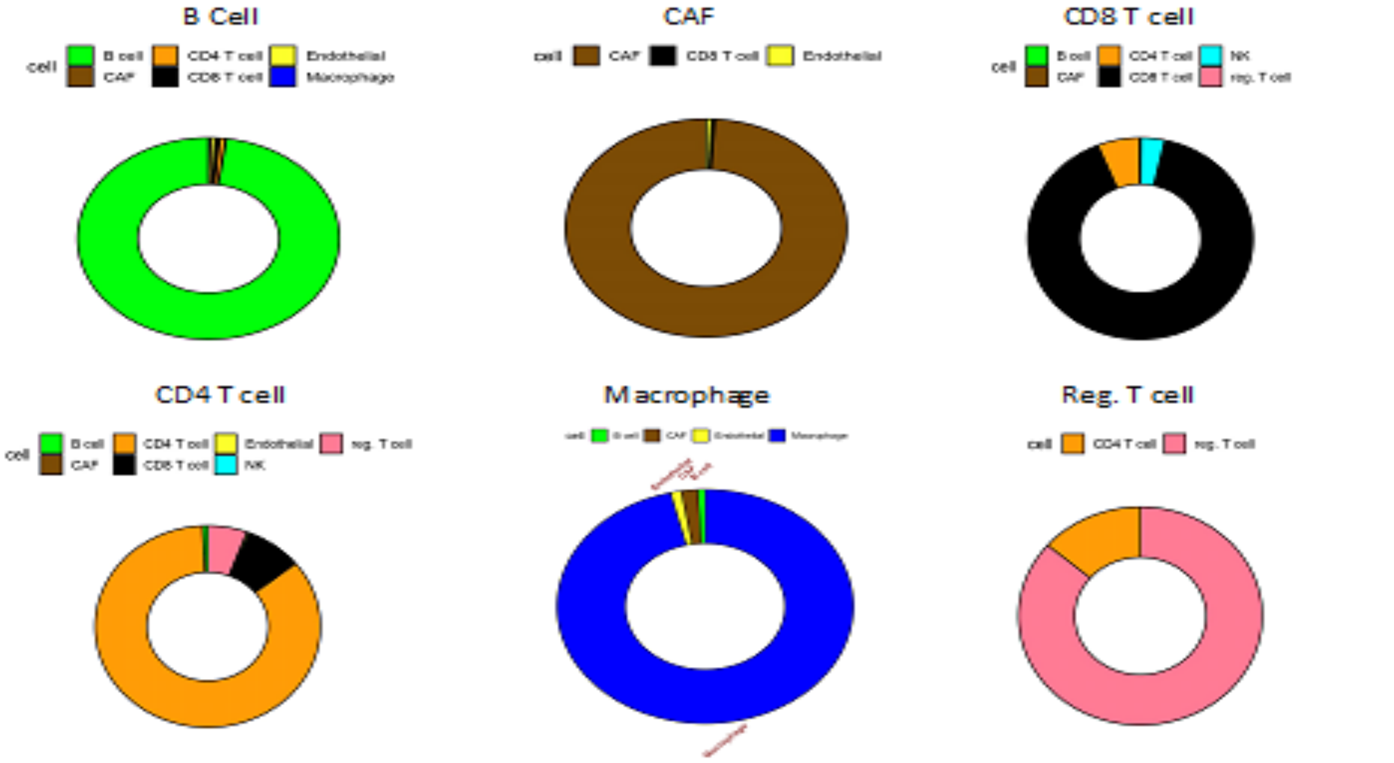

C. Donut plot for some cell types of Goolam Dataset

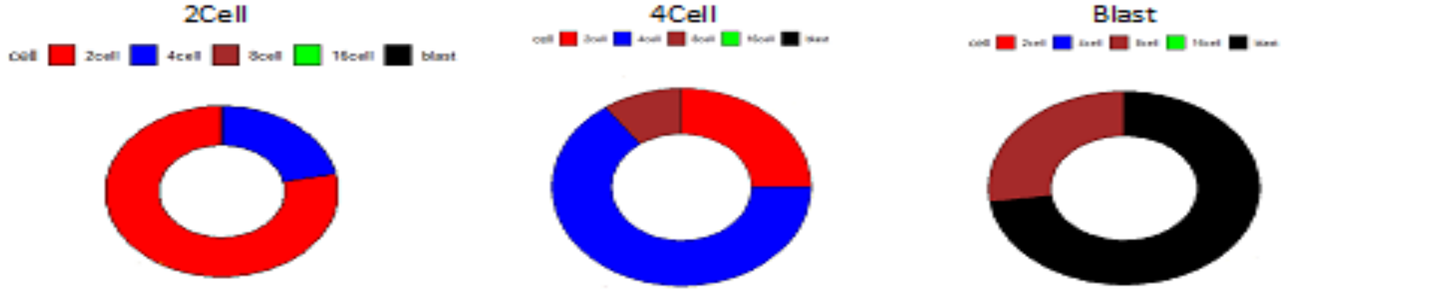

Supplement: Supplementary file 1 [file DataSheet2.PDF]
