# Supplementary figures and images for: A Regularized Multi-Task Learning Approach for Cell Type Detection in Single-Cell RNA Sequencing Data
Source: Front Genet. 2022 Apr 13;13:788832. doi: 10.3389/fgene.2022.788832 (PMC9043858; doi:10.3389/fgene.2022.788832)

Original

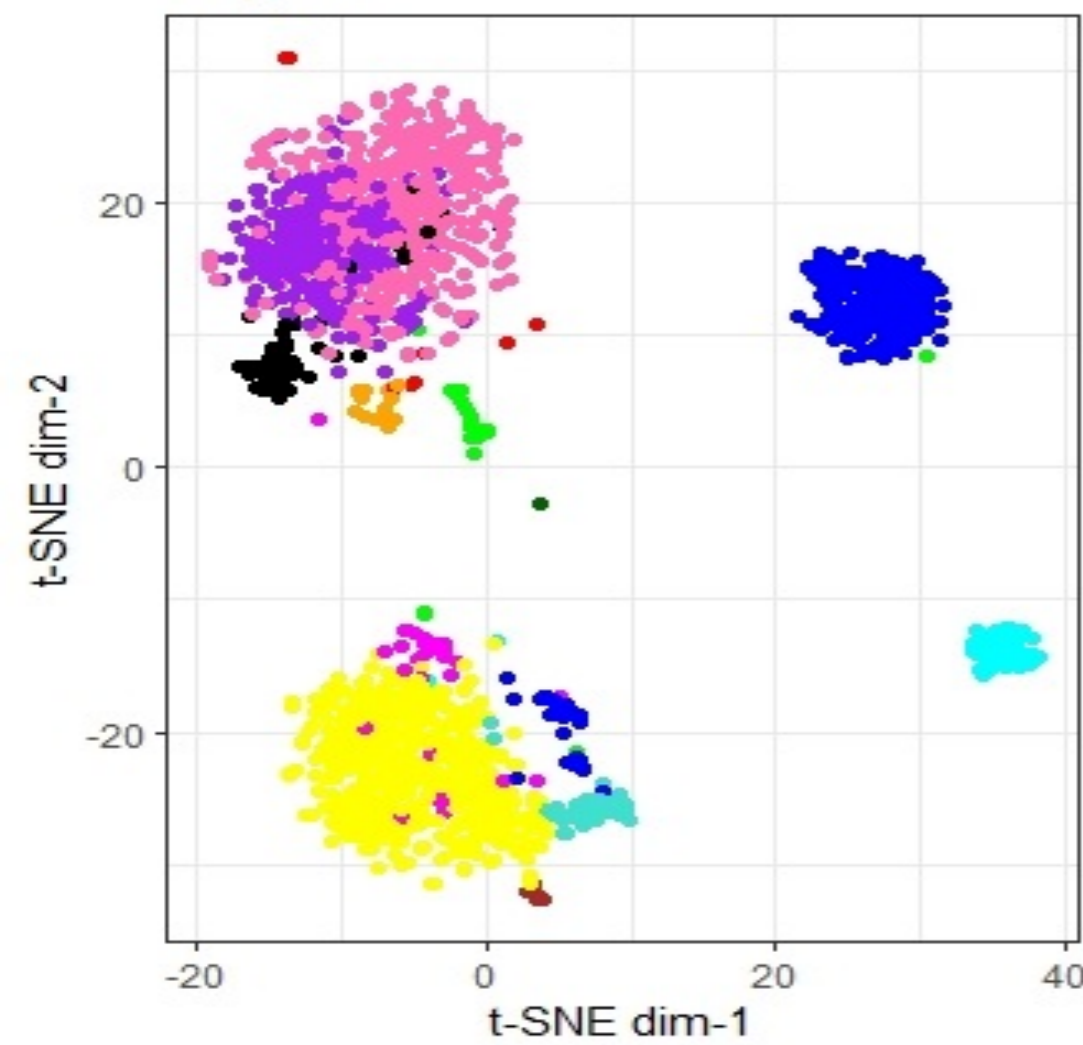

Predicted

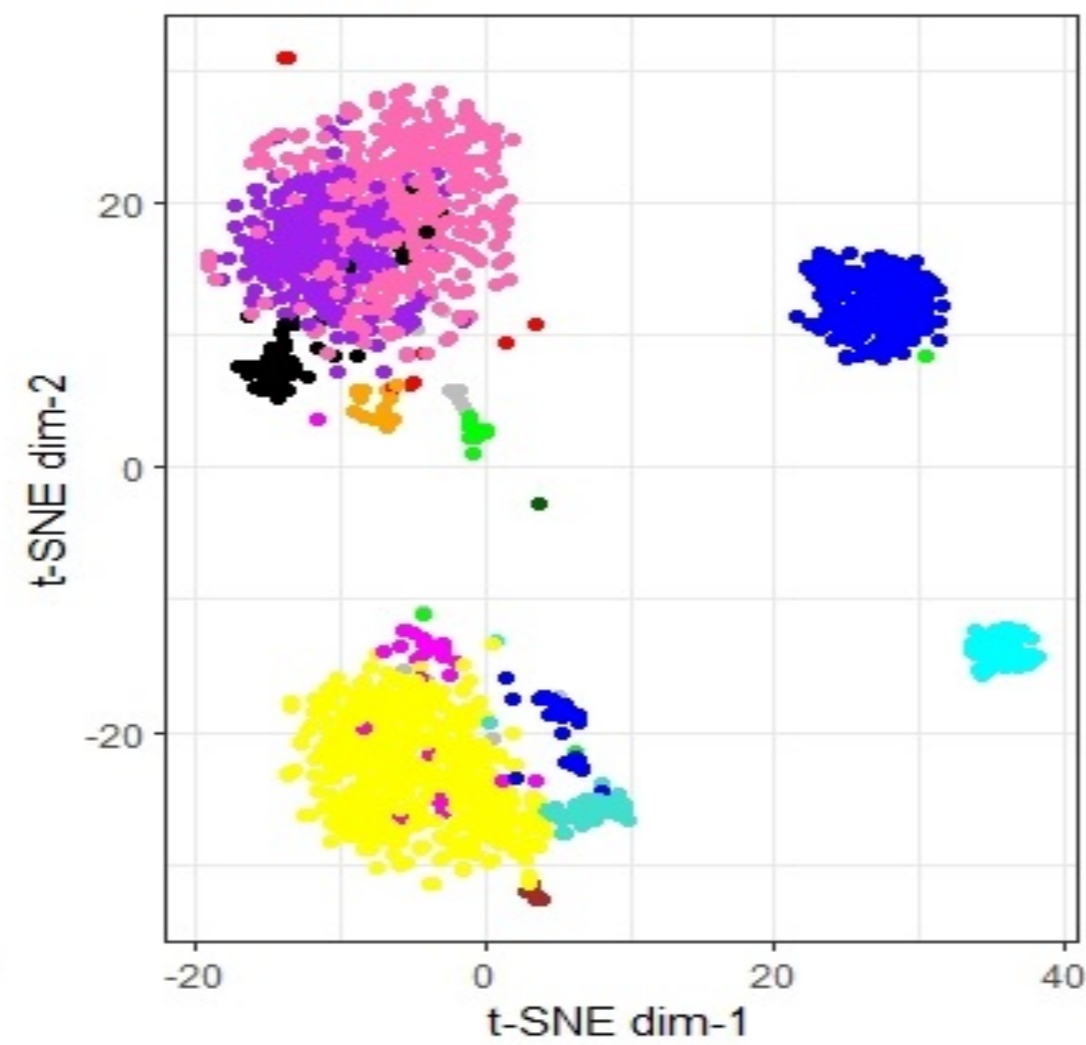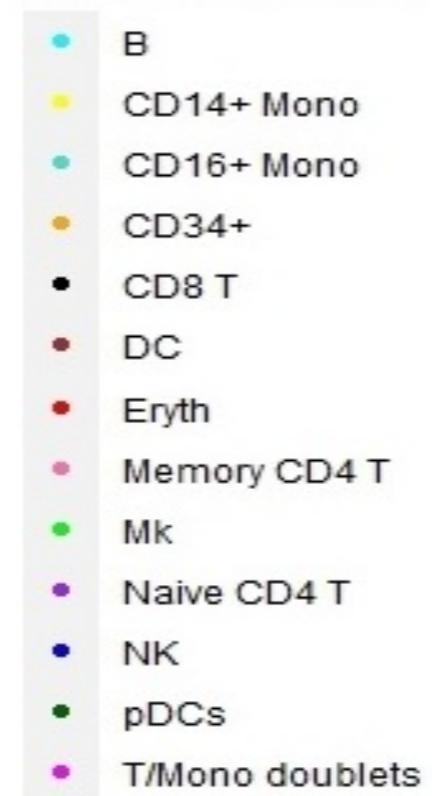

CD34+

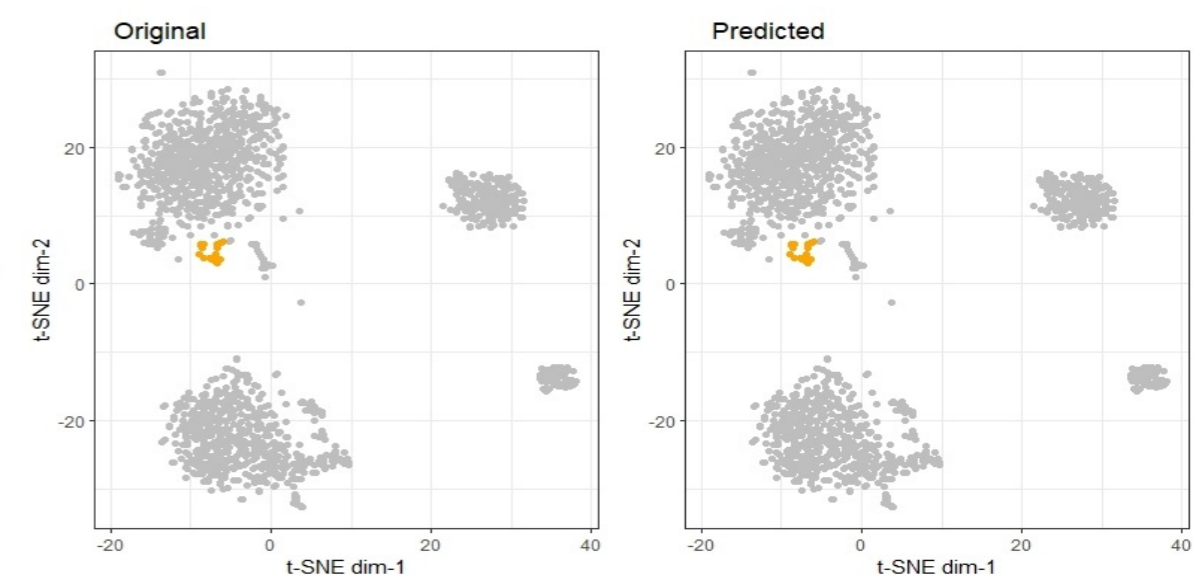

B cell

CD14+ Mono

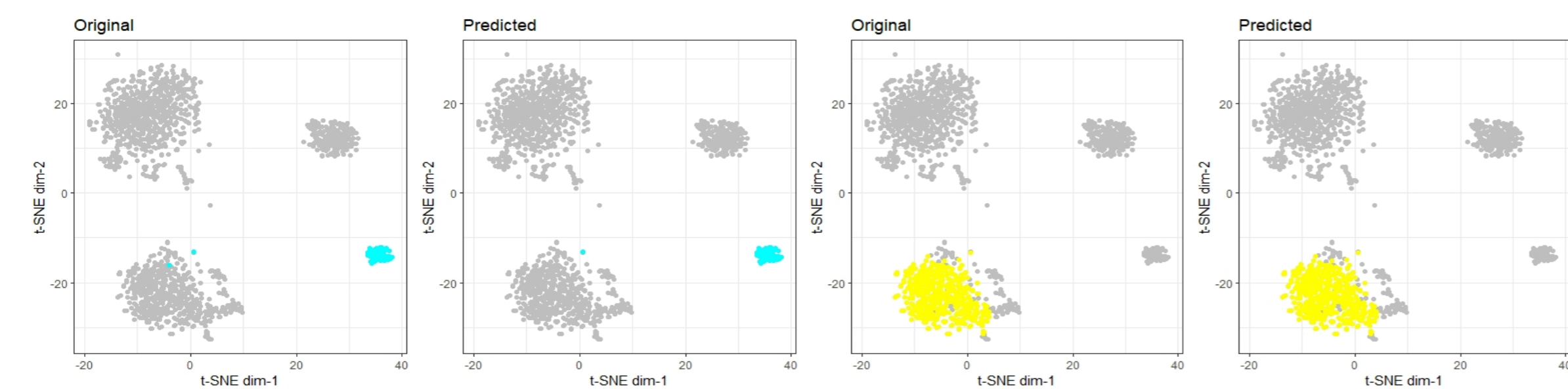

DC

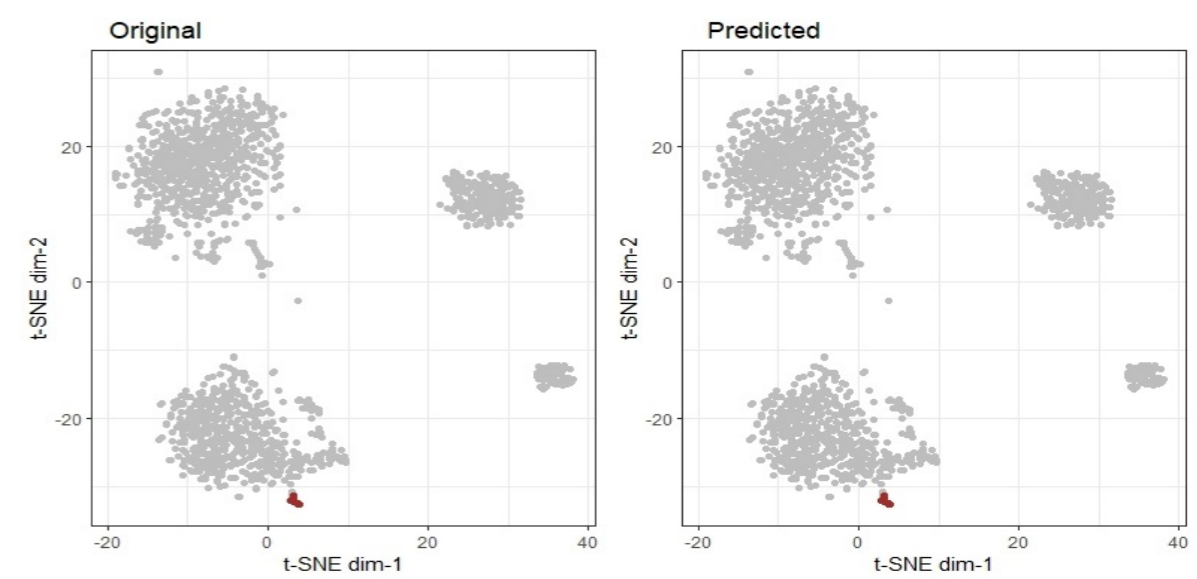

CD16+ Mono

MK

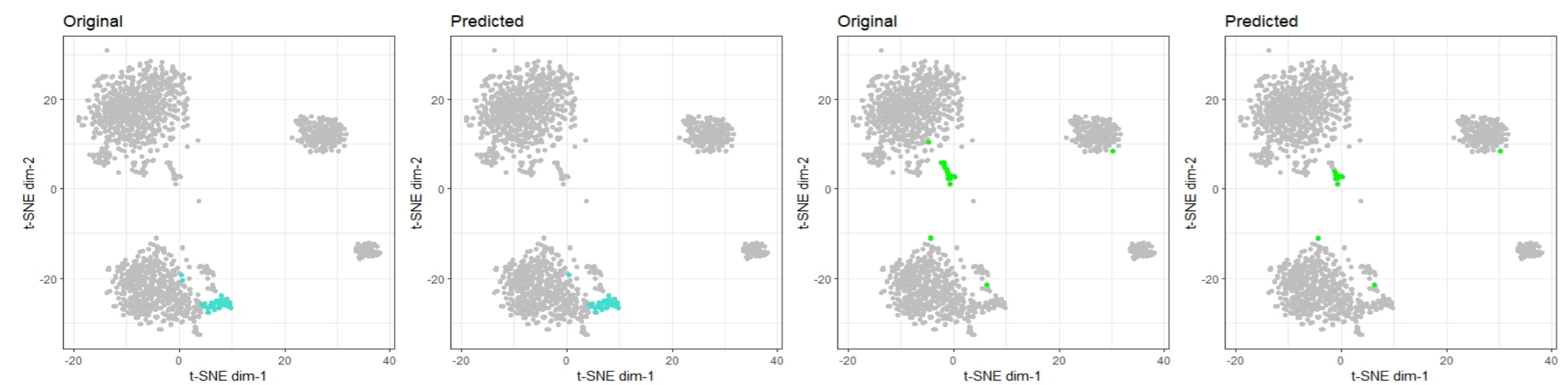

Eryth

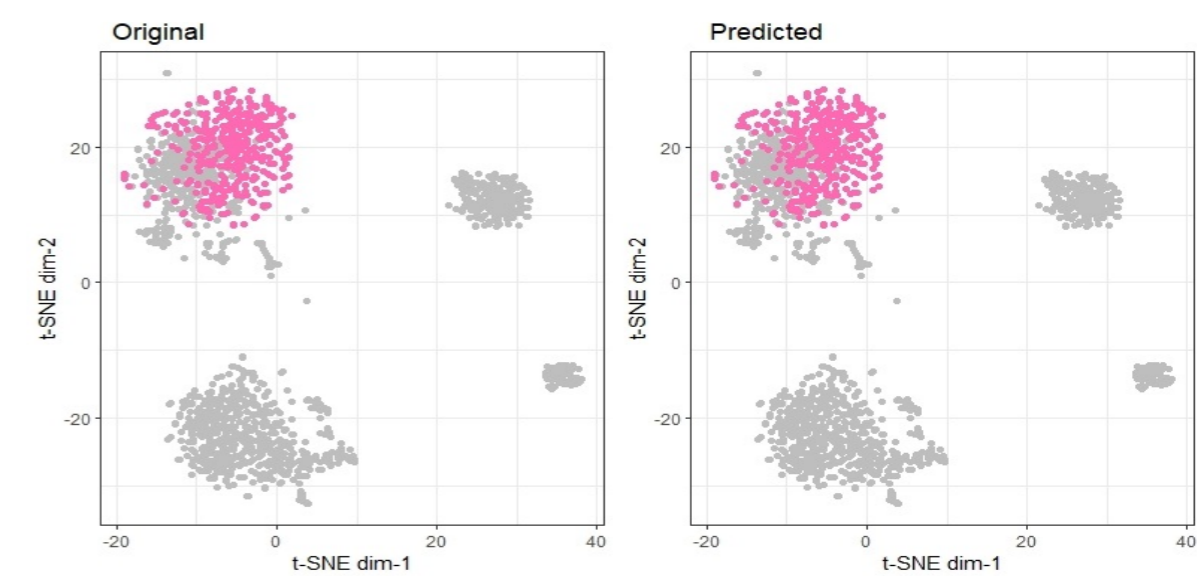

NK

DC

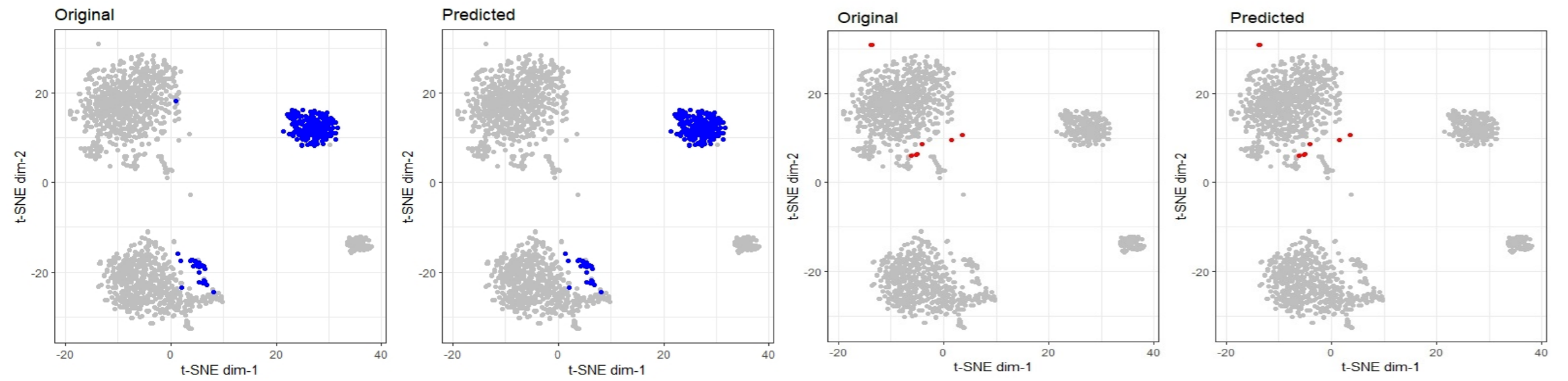

CD8T

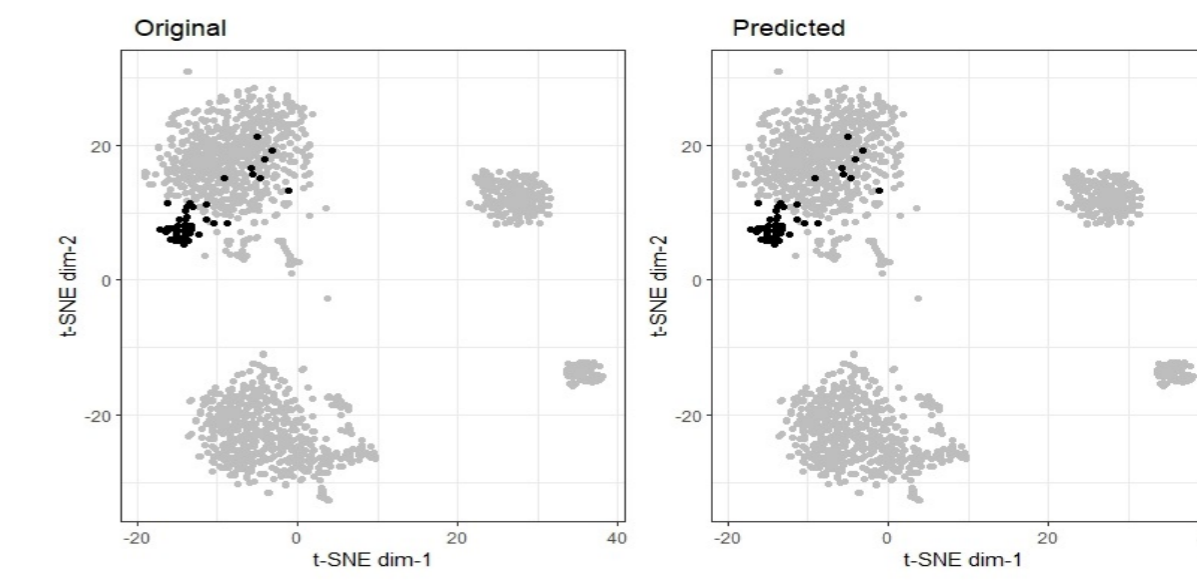

Memory CD4T

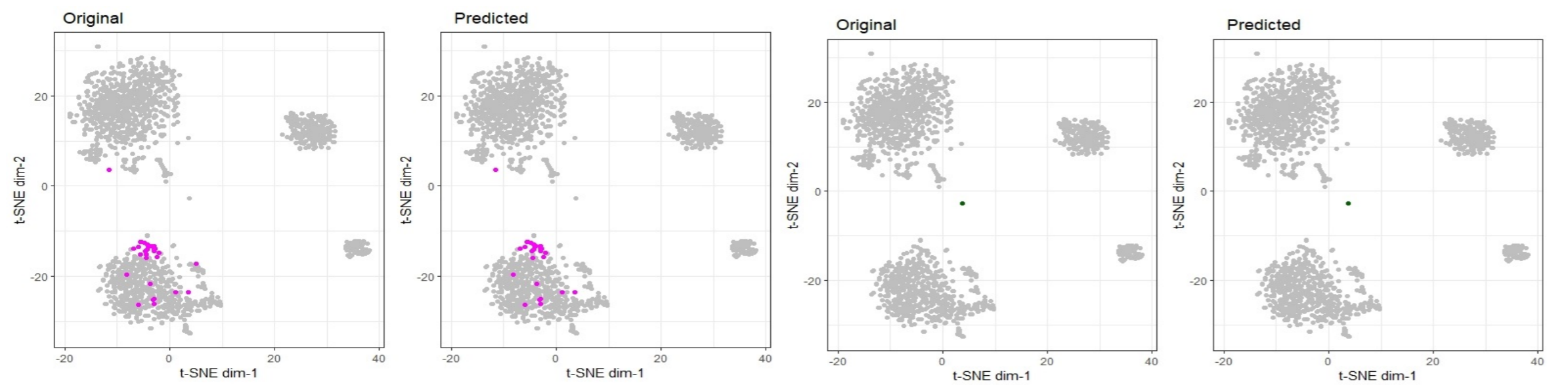

T Mono/doublets

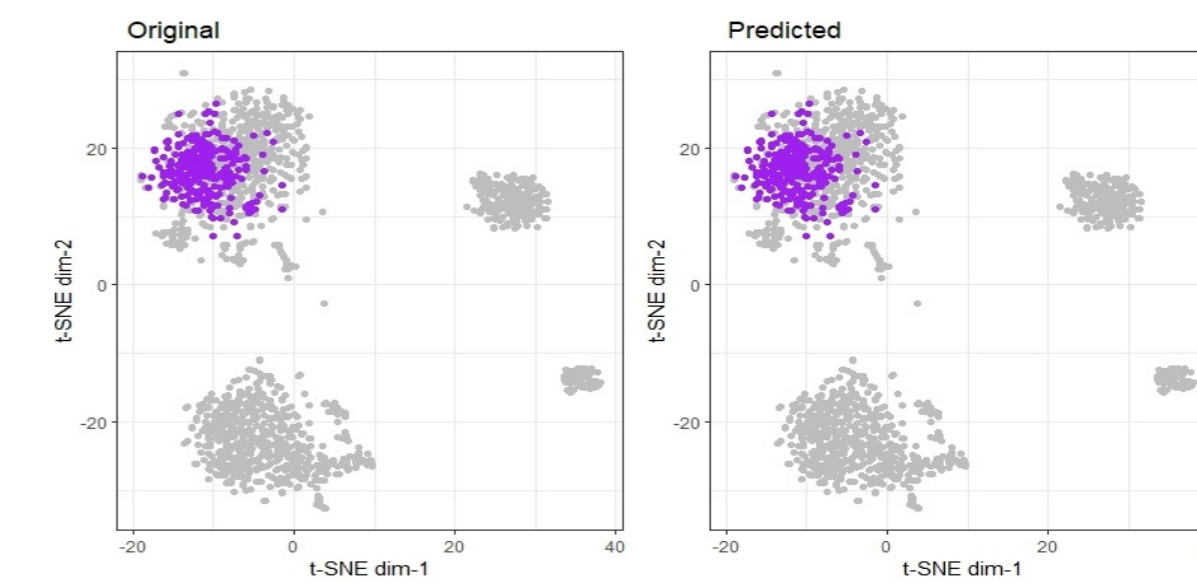

Supplement: Supplementary file 2 [file DataSheet1.PDF]
